# Use of SPY Elite Fluorescence Imaging in Creation of a Continent Urinary Diversion

**DOI:** 10.1155/2019/9069841

**Published:** 2019-12-09

**Authors:** Ali Hajiran, David Zekan, Tyler Trump, Dylan Dangerfield, Adam Luchey

**Affiliations:** ^1^Department of Urology, West Virginia University, Morgantown, WV 26505, USA; ^2^Department of Urology, University of Tennessee Graduate School of Medicine, Knoxville, TN 37920, USA

## Abstract

The use of SPY Elite Fluorescence Imaging has recently grown popular among multiple surgical specialties, including colorectal, plastic, endocrine, ophthalmologic, and vascular surgery, due to its ability to quickly and accurately assess tissue perfusion and guide intraoperative decision making. To our knowledge, the use of SPY imaging in urologic reconstructive surgery has yet to be reported. We present a case in which SPY imaging was used intraoperatively to assess perfusion of an ileocecal anastomosis and a segment of bowel prior to creation of a continent urinary diversion following radical cystectomy.

## 1. Introduction

Inadequate tissue perfusion is a fundamental cause of early complications following surgery [1]. Historically, clinical judgment has been used as the primary means of evaluating tissue perfusion intraoperatively via palpation of pulses, assessing bleeding at cut edges, and observation of tissue color. However, this method is not always reliable. In many cases, more conclusive evaluation of tissue perfusion is desired intraoperatively in order to avoid the potentially devastating complications secondary to tissue ischemia. In urologic reconstructive surgery, ensuring adequate blood supply is critical in the creation of a continent urinary diversion. Failure to identify and address poorly perfused segments of bowel intraoperatively could lead to many devastating complications including anastomotic leak, breakdown, stricture or necrosis of the reservoir [2, 3].

Multiple modalities have been evaluated and used clinically in the past to assess tissue perfusion with mixed results. Use of the SPY Elite Fluorescence Imaging system, which utilizes indocyanine green (ICG) with laser angiography, has grown in popularity in ophthalmologic, plastic, endocrine, vascular, and colorectal surgeries. ICG injected intravenously allows for intraoperative assessment of visceral blood supply and bowel perfusion. This real time assessment allows for increased operative efficiency and assists in surgical decision-making [1]. In this case, we present the use of intraoperative laser angiography to aide in decision making while selecting bowel segment for use as a continent urinary diversion.

## 2. Case Presentation

A 61-year-old woman with high-grade nonmuscle invasive Ta urothelial carcinoma of the bladder presented to our clinic. The patient subsequently experienced recurrence of high-grade disease following two complete induction courses of intravesical BCG; therefore, we recommended proceeding with radical cystectomy. The patient agreed and requested creation of a continent urinary diversion.

**Figure 1:**
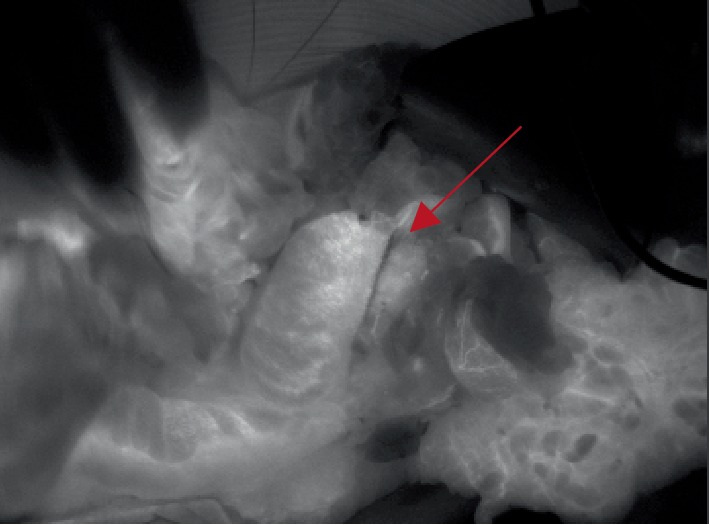
Intraoperative SPY Elite Fluoroscopy Imaging of well-perfused ileocecal anastomosis.

**Figure 2:**
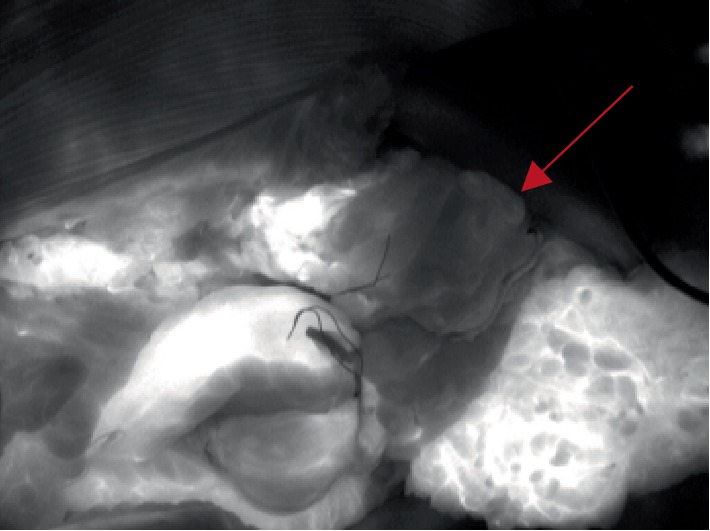
Intraoperative SPY Elite Fluoroscopy Imaging showing poorly perfused segment of ascending colon.

**Figure 3:**
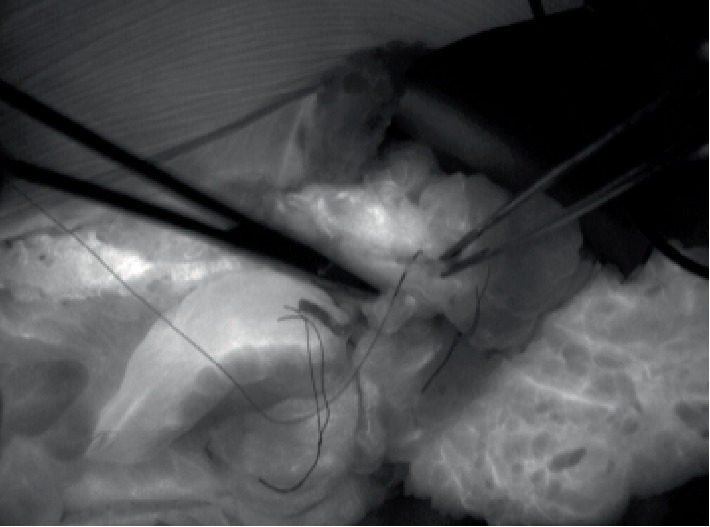
Intraoperative SPY Elite Fluoroscopy Imaging guiding marking of poorly perfused segment of ascending colon with a stitch.

We planned to perform an open radical cystectomy, extended pelvic lymph node dissection, and continent urinary diversion using the ascending colon, terminal ileum, and ileocecal valve as a continence mechanism (Indiana Pouch). Intraoperatively, the right colon was mobilized while carefully preserving the right colic and ileocolic arteries. A 12 cm segment of the distal ileum along with a 30 cm of cecum was harvested using a GIA stapler, while preserving the mesenteric blood supply. Bowel continuity was re-established by creating an ileocolic anastomosis using the GIA stapler. The SPY Elite Fluorescence system then was used to assess adequate perfusion of the anastomosis as well as the harvested bowel to be used for the urinary reservoir. This involved intravenous injection of 1.25–5 mg of a 2.5 mg/mL indocyanine green solution followed by visualization using a laser diode array capable of illuminating a maximum field of 18.5 × 13.5 cm^2^. A device camera then captured these image sequences at a rate of 3.75–30 frames per second based on the desired recording time of 30 second up to a maximum of 4.5 minutes. Images were viewed on a high-definition monitor in real time in the operating room, allowing for immediate evaluation of tissue perfusion [1]. The anastomosis appeared to be well perfused (Figure 1); however, an approximately 3 cm segment of ascending colon demonstrated poor perfusion on SPY imaging (Figure 2). This segment of poorly perfused bowel was marked (Figure 3) and resected to ensure that it was not included in the urinary reservoir. The continent cutaneous urinary reservoir was then created without any complications. The remainder of the surgery was uneventful. The patient's hospital course was uneventful without complications and she was discharged home on post-operative day seven. She followed up three weeks post-operatively for pouchogram, which revealed appropriate filling with no contrast extravasation. Four months later, the patient reported that she was doing very well with no issues performing intermittent catheterization.

## 3. Discussion

Anastomotic leakage and breakdown are serious, potentially fatal complications known to occur in up to 5% of patients undergoing surgery requiring urinary diversion. Several factors can contribute to anastomotic breakdown including poor perfusion, prior radiation therapy, and use of bowel affected by chronic inflammation, among others. With regard to these factors, use of healthy, well-perfused bowel is critical to improving patient outcomes following anastomotic formation [2]. In this case, the authors implement the use of laser angiography to assess the perfusion and viability of bowel segment prior to selection for use in the ureteroenterostomy.

Intraoperative utilization of laser angiography has grown popular over the past decade with proven benefit to intraoperative decision-making and patient outcome in various fields, from mapping of the extrahepatic biliary tree in cholecystectomy to lymphatic mapping in sentinel lymph node biopsy [4]. The investigators in the PILLAR-II trial demonstrated a marked reduction in anastomotic leakage following colorectal surgery in those patients who had intraoperative laser angiography. The incidence of anastomotic breakdown in those who underwent laser angiography was 60% lower than in those who did not, including patients undergoing high-risk bowel anastomoses [5].

While there is sound evidence supporting the use of laser angiography to assist in bowel anastomosis, there is little literature addressing its utility in urologic surgery. Morozov et al. describe the use of SPY elite in lymph node dissection during radical prostatectomy, the identification of renal tumors during partial nephrectomy, and location of stricture during ureteroplasty [6]. Specifically, a majority of malignant renal growths (70%) demonstrated hypofluorescence on imaging, while the remaining group shows no difference in fluorescent uptake [7]. It has also been used in robotic partial nephrectomy to facilitate selective arterial clipping causing regional perfusion deficit directed specifically at a tumor [8]. Another small study demonstrated the benefit of laser angiography in the assessment of ureteral viability prior to ureteroileal anastomosis. In this study, 70% of patients required a segment of distal ureter to be removed prior to spatulation based on poor enhancement during laser angiography [9]. Similarly, Rother et al. demonstrate the utility of intraoperative laser angiography in renal transplant to assess allograft perfusion following implantation of the donor kidney [10]. The future of fluorescent imaging in urology is vast, with current developments describing the use of tumor markers such as prostate-specific membrane antigen with fluorescent tracers for intraoperative imaging in urologic oncology as well as diagnosis of shallow papillary bladder carcinoma and carcinoma in situ using fluorescent markers with high sensitivity and specificity [11, 12]. This case provides more supporting evidence of the benefits associated with the use of SPY technology in urology. This is the first case describing its use to aid in the formation of a successful neobladder.

## 4. Conclusion

The authors of the case described above present the use of laser angiography as an effective method to assist in selection of bowel for use during urinary diversion. We expect that this work will support fellow clinicians and researchers to explore laser angiography as a means for evaluating tissue viability. Further studies would be helpful in providing further support of low anastomotic leak and necrosis.
